# *In vitro* impact of *Streptococcus mitis* on the inhibition of oral cancer cell proliferation via mitotic modulation

**DOI:** 10.3389/fcimb.2025.1524820

**Published:** 2025-05-09

**Authors:** Inori Inui, Shinichi Mochizuki, Fumika Hirabayashi-Nishimuta, Yoshie Yoshioka, Osamu Takahashi, Masaaki Sasaguri, Manabu Habu, Wataru Ariyoshi, Ryota Yamasaki

**Affiliations:** ^1^ Division of Infections and Molecular Biology, Department of Health Promotion, Kyushu Dental University, Kitakyushu, Fukuoka, Japan; ^2^ Division of Maxillofacial Surgery, Department of Science of Physical Functions, Kyushu Dental University, Kitakyushu, Fukuoka, Japan; ^3^ Department of Chemistry and Biochemistry, The University of Kitakyushu, Kitakyushu, Fukuoka, Japan; ^4^ Division of Oral Medicine, Department of Science of Physical Functions, Kyushu Dental University, Kitakyushu, Fukuoka, Japan; ^5^ Collaborative Research Centre for Green Materials on Environmental Technology, Kyushu Institute of Technology, Kitakyushu, Fukuoka, Japan

**Keywords:** oral microbiota, *Streptococcus* spp., oral cancer, carcinoma, cell cycle, DUSP1, mitotic nuclear division, *Streptococcus mitis*

## Abstract

**Introduction:**

Recent studies have elucidated a potential correlation between oral carcinogenesis and the oral microbiome. However, few reports exist on the interaction between *Streptococcus* spp., the most common oral microflora bacterium, and oral cancer. In this study, we aimed to elucidate the effects of *Streptococcus* spp. on oral squamous cell carcinoma (OSCC) cells *in vitro*.

**Methods:**

HSC-3 (tongue carcinoma) and Ca9-22 (gingival carcinoma) cells were used as models of OSCC cells, and their responses were examined after adding major oral *Streptococcus* species—*S. mitis*, *S. sanguinis*, *S. anginosus*, *S. salivarius*, and *S. mutans*—to the culture medium. Cell viability was assessed using the CCK-8 assay. Gene expression changes were analyzed using RNA sequencing and RT-qPCR followed by Gene Ontology analysis. Flow cytometry was used to observe the effects of bacteria on the cell cycle.

**Results:**

Among all examined *Streptococcus* species, *S. mitis* had the strongest inhibitory effect on the growth of OSCC cells. RNA sequencing and RT-qPCR revealed an increase in the number of genes involved in mitotic nuclear division, especially *DUSP1*, in HSC-3 cells treated with *S. mitis.* Flow cytometry showed that *S. mitis* caused a decreased number of HSC-3 cells in the G0/G1 phase and an increased number in the G2/M phase, suggesting cell cycle arrest in the G2/M phase. Various treatments of *S. mitis* were used to examine the effects of intact bacteria and bacterial components on cancer cells, indicating the involvement of structural bacterial proteins.

**Conclusions:**

This study, investigating the association between oral cancer cells and bacteria of the genus *Streptococcus*, revealed that *S. mitis* may play an important role in the inhibition of cancer cells.

## 1 Introduction

Oral cancer encompasses a group of malignant neoplasms localized within the jaw and oral cavity. As the mucosa consists of squamous epithelium covering the entire oral cavity apart from the teeth, 90% of all oral cancer cases are classified as squamous cell carcinoma (Bagan et al., 2010). In 2020, 377,713 cases of oral cancer and 177,757 related deaths were reported worldwide (Sung et al., 2021). According to the Global Cancer Observatory (GCO), the incidence of oral squamous cell carcinoma (OSCC) is projected to rise by approximately 40% by 2040, with increasing mortality rates (Tan et al., 2023). The primary treatment for oral cancer is surgical resection, but tissue loss from surgery can affect the dentition, muscles, and nerves. Since these tissues are involved in important functions, such as chewing, swallowing, and speech, the quality of life after treatment is significantly reduced in cases that require extensive resection (Vermaire et al., 2022).

In addition to smoking, alcohol consumption, and improper oral hygiene, various systemic predisposing factors such as nutritional deficiencies, immunodeficiency, and genetic disorders are intricately involved as major risk factors in the pathogenesis of oral cancer (Chamoli et al., 2021; Tan et al., 2023). The mechanisms of pathogenesis also vary, and unidentified risk factors may exist. Cancer is a multifactorial disease caused by various genetic abnormalities (Hanahan and Weinberg, 2011), and its pathogenesis can involve the induction of an oncogenic inflammatory environment, DNA damage, and the production of molecules involved in tumorigenic signaling by the surrounding bacterial flora (Plottel and Blaser, 2011; Cho and Blaser, 2012). *Helicobacter pylori* has been found to cause gastric cancer (Polk and Peek, 2010); since this discovery, studies have also focused on the involvement of specific microorganisms in cancer development. Examples include typhoid bacteria as a possible risk factor for gallbladder cancer (Espinoza et al., 2016) and human papillomavirus (HPV) being associated with cancers at various sites, including the cervix and mid-pharynx (Egawa, 2023). Furthermore, recent studies have demonstrated that oral microbiota are involved in tumor development and progression (Sun et al., 2020). In addition to the involvement of HPV (Sun et al., 2020) and *Candida albicans* (Wang et al., 2023), the major periodontopathogenic bacteria *Fusobacterium nucleatum* and *Porphyromonas gingivalis* are particularly relevant. These bacteria trigger excessive inflammatory responses, evade the immune system, have anti-apoptotic activity, cause cellular transformation, and promote cancer (Karpiński, 2019; Li et al., 2023).

More than 700 species of bacteria are present in the oral cavity and play an important role in maintaining a healthy oral environment by forming complex commensal flora; however, some bacteria are pathogenic and can cause oral diseases. Among the commensal bacteria inhabiting the oral cavity, *Streptococcus* is the most abundant at the genus level (Costalonga and Herzberg, 2014). Bacteria of the genus *Streptococcus* can exhibit both beneficial effects and pathogenicity in the oral cavity. While *Streptococcus salivarius* and *Streptococcus mitis* inhibit the growth of pathogenic bacteria, *Streptococcus sanguinis* and *S. mitis* are known to cause infective endocarditis (Okahashi et al., 2022; Jenkinson, 2011). *Streptococcus anginosus* is a causative agent of odontogenic infections (Asam and Spellerberg, 2014), whereas *Streptococcus mutans* and *Streptococcus sobrinus* metabolize sugars to produce acid, contributing to dental caries (Jenkinson, 2011). Thus, *Streptococcus* species are closely associated with other microorganisms and various diseases; however, their relationship with oral cancer remains largely unknown. In a previous study, saliva from patients with OSCC lesions contained more *Capnocytophaga gingivalis*, *Prevotella melaninogenica*, and *S. mitis* than saliva from cancer-free individuals, although the reasons for this difference are unclear. As mentioned above, the interaction of *F. nucleatum* and *P. gingivalis* with oral cancer has been demonstrated. However, the influence of *Streptococcus* spp., the most abundant genus in the oral cavity, on OSCC remains unclear, and its exact role in the pathogenesis of cancer remains unknown, although the association of the latter with *S. anginosus* has been pointed out (Sasaki et al., 2005). Reports on other *Streptococcus* species are also limited. Considering that the effects of the main components of oral flora on oral cancer should be taken into account, the present study aimed to elucidate the effects of *Streptococcus* spp. on oral cancer cells *in vitro.*

## 2 Materials and methods

### 2.1 Cell and bacterial culture

The OSCC strains HSC-3 (from human tongue carcinoma) and Ca9-22 (from human gingival carcinoma) were obtained from the Japanese Collection of Research Bioresources (JCRB; Osaka, Japan). HSC-3 cells were cultured in Eagle’s Minimum Essential Medium (E-MEM; FUJIFILM Wako Pure Chemical Co., Osaka, Japan) supplemented with 10% fetal bovine serum (FBS) and 1% penicillin/streptomycin (SMPC). Ca9-22 cells were cultured in Minimum Essential Medium α (MEMα; FUJIFILM Wako Pure Chemical Co.) with 10% FBS and 1% SMPC. Both strains were incubated at 37°C in a 5% CO_2_ environment.

The model bacterial strains used were *S. mitis* ATCC 49456, *S. sanguinis* ATCC 10556, *S. anginosus* ATCC 33379, *S. salivarius* ATCC BAA- 1024, and *S. mutans* UA 159. Brain Heart Infusion (BHI; BD, Franklin Lakes, NJ) agar medium containing 1% yeast extract (BD) was used for all bacterial cultures, and the bacteria were incubated for 1 to 2 days at 37°C in a 5% CO_2_ environment. Single colonies were seeded on BHI liquid medium and incubated for 12 h at 37°C, 5% CO_2_. The culture medium (5 mL) was prepared to achieve a turbidity of 0.4 at 600 nm, and all precipitates were resuspended in an equal volume of E-MEM or MEMα with FBS and 1% SMPC after centrifugation at 3500 × *g* for 10 min. The centrifugation and resuspension processes were repeated twice to wash the pellets. After washing, the colony-forming units (CFUs) were counted by plating the culture onto BHI agar.

### 2.2 Cell viability assay

Cell viability was assessed using a Cell Counting Kit-8 with WST-8 (CCK-8; Dojin Chemical Laboratory, Kumamoto, Japan). Briefly, HSC-3 and Ca9-22 cells were cultured and adhered in 96-well plates at 2000 cells/well for 6 h. Each bacterial solution, resuspended in cell culture medium, was prepared 100–0.1 v/v% by 2-fold serial dilution in a separate 96-well plate. Then 50 μL of each bacterial solution was added to 50 μL of cancer cells (100 μL total). After 48 h of incubation, 10 µL of CCK-8 was added and incubated at 37°C for 2 h. The absorbance of each well at 450 nm was measured using a microplate reader (Multiskan FD, Thermo Fisher Scientific, Waltham, MA). All experiments were conducted with at least three biological replicates.

### 2.3 Changes in gene expression

Total RNA was extracted from each sample using the Cica Geneus RNA Prep Kit (for tissue) (Kanto Chemical, Tokyo, Japan). After culturing the cells for 48 h in the presence of bacterial solution, RNA was extracted. The quality and quantity (concentration ≥ 40 ng/μL, A260/A230 ≥ 2.0, and A260/A280 = 1.8–2.2) were evaluated using a Nanodrop (Thermo Fisher Scientific). Total RNA was provided to Novogene (China) for RNA sequencing using Nova Seq 6000 (Illumina Inc., San Diego, CA, USA). The raw data in FASTQ format were processed using Novogene’s Perl script and cleaned by removing adapter-containing reads, poly-N-containing reads, and low-quality reads. The reference genome index was constructed using Hisat2 v2.0.5, and paired-end clean 1 read were aligned to the reference genome using Hisat2 v2.0.5. Gene expression levels were quantified using featureCounts v1.5.0-p3. Differential expression was analyzed using the DESeq2R package (1.20.0). Genes with adjusted *p*-values of < 0.05 in DESeq2 were considered differentially expressed genes. Gene Ontology (GO) functional analysis was performed using the clusterProfiler R package with gene length bias correction. GO terms with adjusted *p*-values of < 0.05 were considered to have significantly changed functions due to differentially expressed genes. For real-time RT-qPCR, RNA was reverse-transcribed and amplified according to previously described methods (Ariyoshi et al., 2017). RNA expression was analyzed by real-time RT-qPCR using the AriaMx Real-Time PCR system (Agilent Technologies, Santa Clara, CA). The primer sequences used for RT-qPCR are listed in **Table 1**.

**Table 1:** The primer sequences used for RT-qPCR.

Gene name		Primer sequence (5′-3′)
*GAPDH*	Forward	GAC GGC CGC ATC TTC TTG T
	Reverse	CAC ACC GAC CTT CAC CAT TTT
*DUSP1*	Forward	CCA TCT GCC TTG CTT ACC TTA T
	Reverse	GCT GAA GTT GGG AGA GAT GAT G

### 2.4 Flow cytometry analysis of bacterial effects on the cell cycle

After a 6 h pre-culture, 7 × 10^5^ HSC-3 cells were seeded per Petri dish (10 cm in diameter) for 24 h. Then, the *S. mitis* bacterial solution was added, and the cells were fixed in 70% ethanol for 2 h. The fixed cells were treated with PI/RNase solution (IMMUNOSTEP, Salamanca, Spain). The fluorescence intensity of the cells was observed using a flow cytometer (CytoFLEX; Beckman Coulter, Brea, CA).

### 2.5 Effects of intact bacteria and bacterial components on cancer cells

Live bacteria, secretions of live bacteria, and dead bacteria (sonicated and treated with isopropanol) were used as samples. Culture supernatants were prepared to achieve an optical density of 0.4 at 600 nm of the bacterial culture medium, and the supernatant fluid after centrifugation was extracted by passing it through a 0.2 µm filter. Samples of dead bacteria were obtained by treating live bacteria with 70% isopropanol for 1 h. The bacterial solution was sonicated using an ultrasonic homogenizer (SFX150, Branson Ultrasonics, Brookfield, CT) at 15-second intervals for 5 min, and the solution containing the disrupted bacterial cells was further centrifuged (3500 × *g* for 10 min) to separate the internal components of the bacteria (supernatant) from other bacterial structures (precipitate). The sonicated bacteria were treated with Proteinase K at 65°C for 2 h, then the protease was inactivated at 98°C for 10 min to prepare samples of the bacterial components excluding proteins. Each bacterial solution was added to HSC-3 cells for the cell viability assay. The LIVE/DEAD Viability/Cytotoxicity Kit (Thermo Fisher Scientific) was used to stain live and dead bacteria (sonicated and treated with isopropanol) to observe them under a fluorescence microscope.

### 2.6 Statistical analysis

All data were expressed as mean ± standard deviation (SD). Student’s t-test was used for comparisons between two groups using Microsoft Excel. For comparisons between three or more groups, the statistical analysis software EZR was used to perform one-way analysis of variance (ANOVA) with Tukey’s *post hoc* test.

## 3 Results

### 3.1 *Streptococcus mitis* most effectively inhibits cancer cell growth

**Figure 1:**
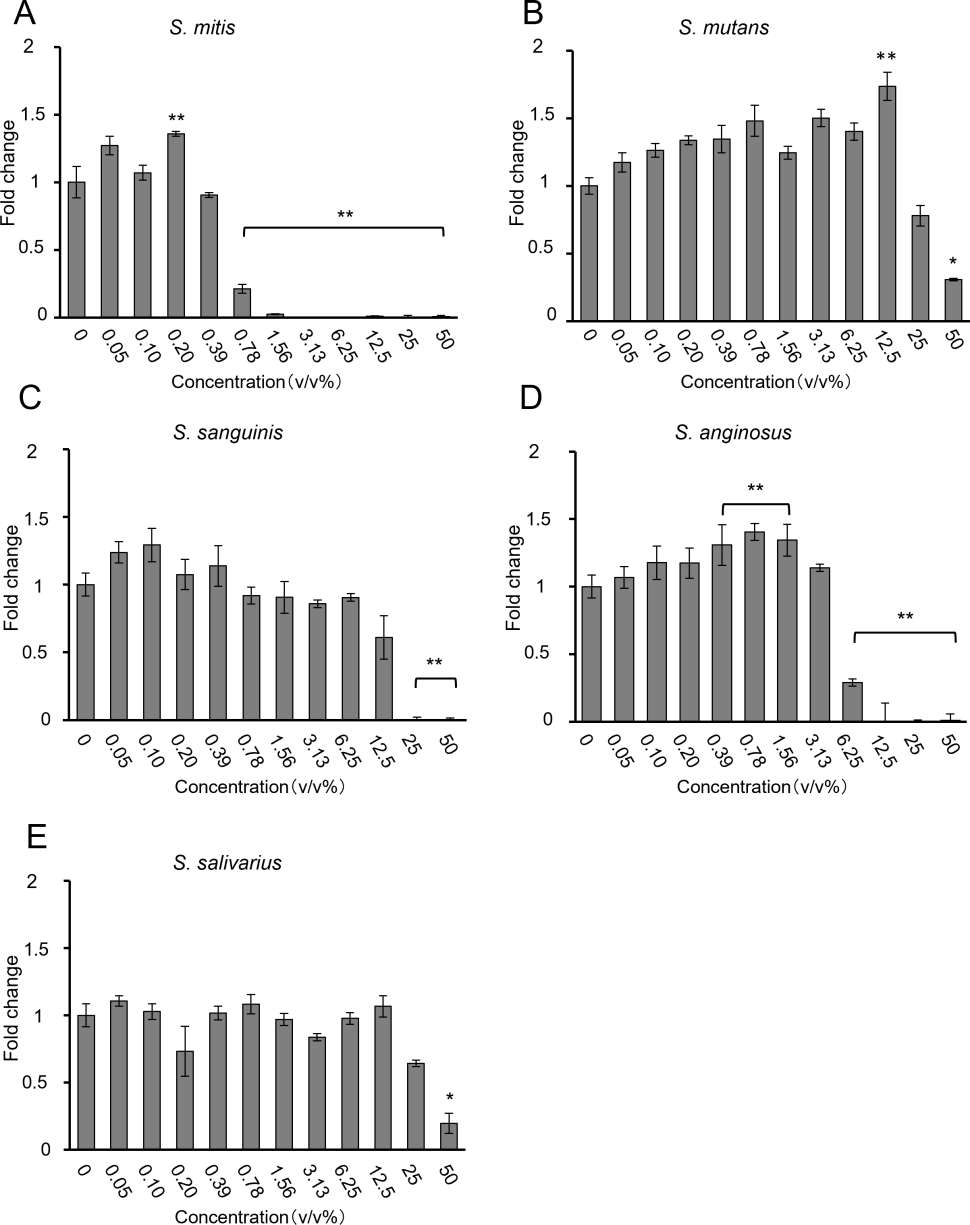
Proliferation of HSC-3 cells, as assessed using the CCK-8 assay, when **(A)** *S. mitis*, **(B)** *S. mutans*, **(C)** *S. sanguinis*, **(D)** *S. anginosus*, and **(E)** *S. salivarius* were added (n = 3). The vertical axis represents the bacterial solution concentration (v/v%), and the horizontal axis shows the fold change. *p < 0.05, **p < 0.01 (Tukey’s test after one-way analysis of variance).

To compare the effects of different bacterial species, bacterial solutions of *S. mitis*, *S. mutans*, *S. sanguinis*, *S. anginosus*, and *S. salivarius* were added for 48 h to HSC-3 cells pre-cultured two-dimensionally in 96-well plates. The number of viable cells was evaluated using the CCK-8 assay. The results are shown in **Figures 1A–E**.

**Figure 2:**
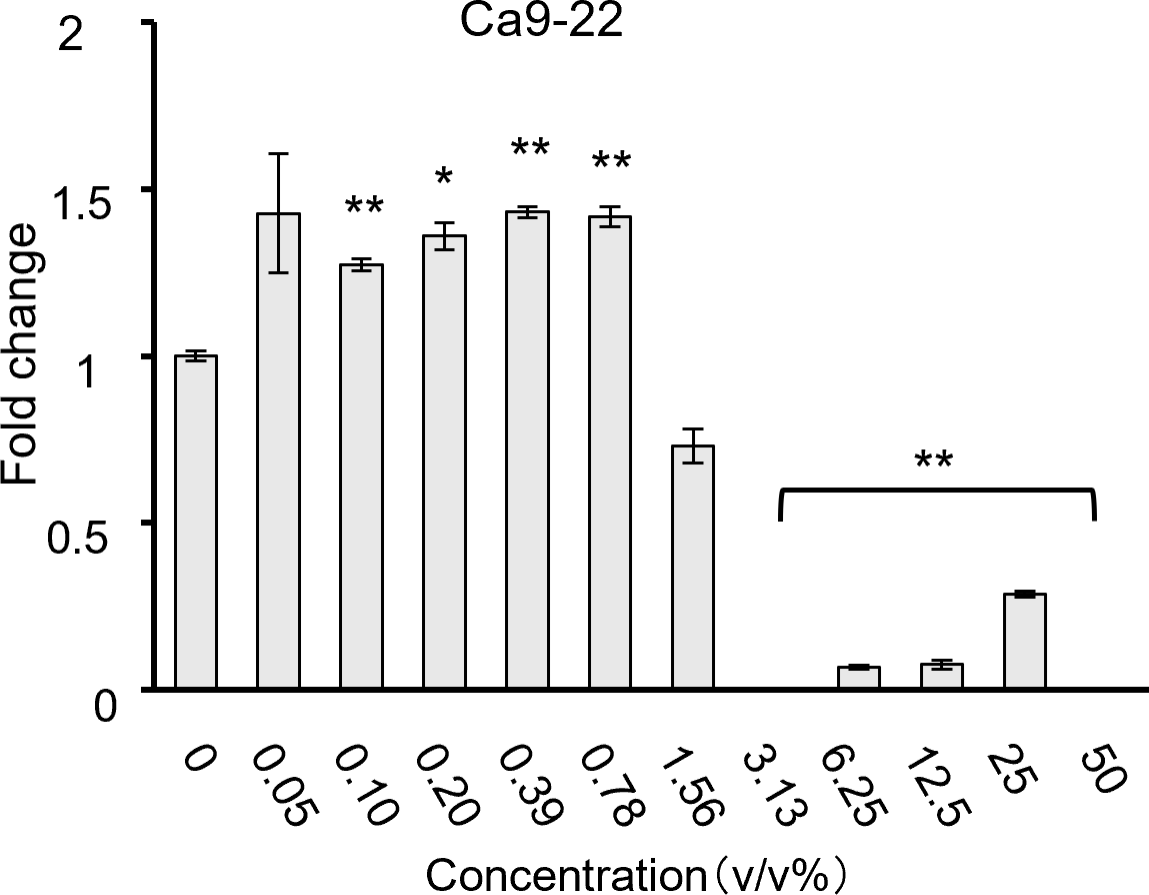
Cell proliferation when *S. mitis* bacteria were added to Ca9-22 cells, as assessed using the CCK-8 assay (n = 3). The vertical axis represents the concentration of bacterial solution (v/v%), and the horizontal axis shows the fold change. *p < 0.05, **p < 0.01 (Tukey’s test after one-way analysis of variance).

Most bacteria inhibited HSC-3 growth in a dose-dependent manner. *S. mitis* significantly inhibited growth at concentrations above 0.78% (**Figure 1A**); *S. mutans and S. salivarius* significantly inhibited only at the highest concentration of 50% (**Figures 1B, E**); *S. sanguinis* significantly inhibited at concentrations of > 25% (**Figure 1C**); and *S. anginosus* significantly inhibited cell growth at concentrations of > 6.25% (**Figure 1D**). The concentrations at which cancer cells were inhibited by 80% (IC80) compared to the control group (0%) are shown in **Table 2**. Among these, *S. mitis* showed the highest inhibition of cancer cell growth. The results of the WST-8 assay when *S. mitis* was added to Ca9-22 cells are shown in **Figure 2**. Although the sensitivity of Ca9-22 cells was inferior to that of HSC-3 cells, significant inhibition of cell proliferation was observed with bacterial concentrations above 3.13%.

**Table 2:** IC80 of HSC-3 cells following the addition of *S. mitis*, *S. mutans*, *S. sanguinis*, *S. anginosus*, and *S. salivarius*.

Bacteria	IC80 (v/v%)
*S. mitis*	0.78
*S. mutans*	–
*S. sanguinis*	25
*S. anginosus*	12.5
*S. salivarius*	50

### 3.2 A group of genes involved in mitosis and nuclear division

**Figure 3:**
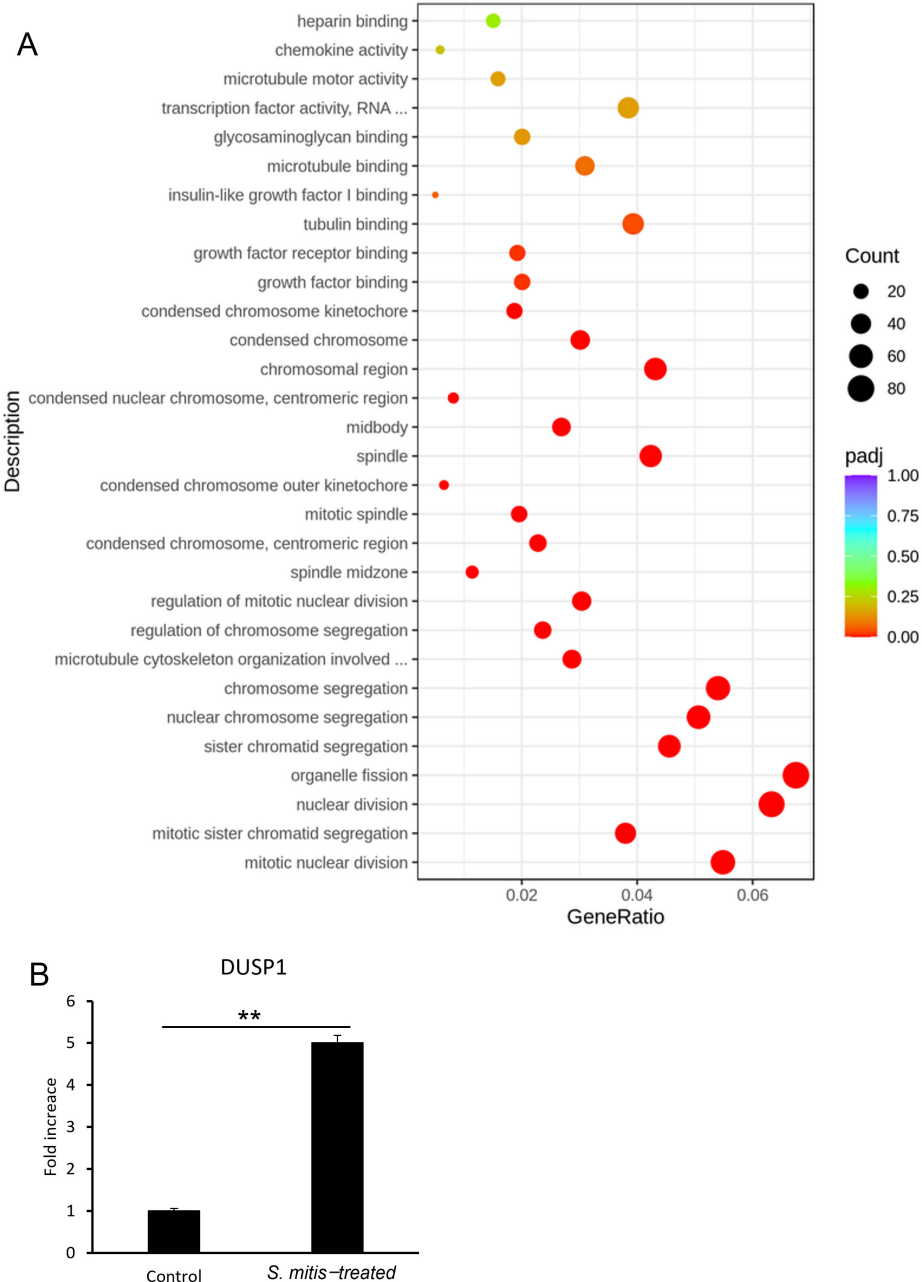
**(A)** Gene expression in HSC-3 cells treated with *S. mitis.* The vertical axis shows the most significant GO term from the bottom up. The horizontal axis shows the ratio of the number of differentially expressed genes linked to the GO term to the total number of differentially expressed genes. The size of the points indicates the number of genes annotated to a specific GO term, and the color indicates the significant level of enrichment. **(B)** *DUSP1* mRNA levels in HSC-3 cells treated with *S. mitis* (n = 3). *p < 0.05, **p < 0.01 (Student’s t-test).

*S. mitis* was added to HSC-3 cells at 1.56%, a concentration that significantly inhibited HSC-3 cell growth (see section 3.1), and RNA was extracted from these cells. The CFU of *S. mitis* at 1.56% was 3.8 × 10^6^ CFU/mL. The multiplicity of infection (MOI) was calculated to be 190. The effects of the bacterial species on gene expression were verified via RNA sequencing. The results of the GO enrichment analysis are shown in **Figure 3A** where the 30 most important GO terms are displayed. The color represents the level of significance, and the dot size represents the number of genes. The number of genes involved in mitosis and nuclear division was significantly increased in the group incubated with *S. mitis* (**Figure 3A**). The specific genes included in this gene group are shown in **Supplementary Figure 1**. DUSP1 expression, which satisfied the conditions |log2(Fold Change) | ≥ 1 and adjusted p ≤ 0.05, was validated by RT-qPCR (**Figure 3B**).

The results showed a significant increase in *DUSP1* gene expression in the *S. mitis-added* group, which was consistent with the RNA sequencing results. In addition, the directed acyclic graph of biological processes allowed us to visualize the enriched GO terms of differentially expressed genes and their hierarchy (**Supplementary Figure 2**). The functional range in this figure becomes more specific from top to bottom. Given the presence of cell cycle genes high up in this hierarchy, we next investigated the cell cycle of stimulated cancer cells.

### 3.3 *S. mitis* treatment decreases the percentage of HSC-3 cells in the G0/G1 phase and increases that in the G2/M phase

**Figure 4:**
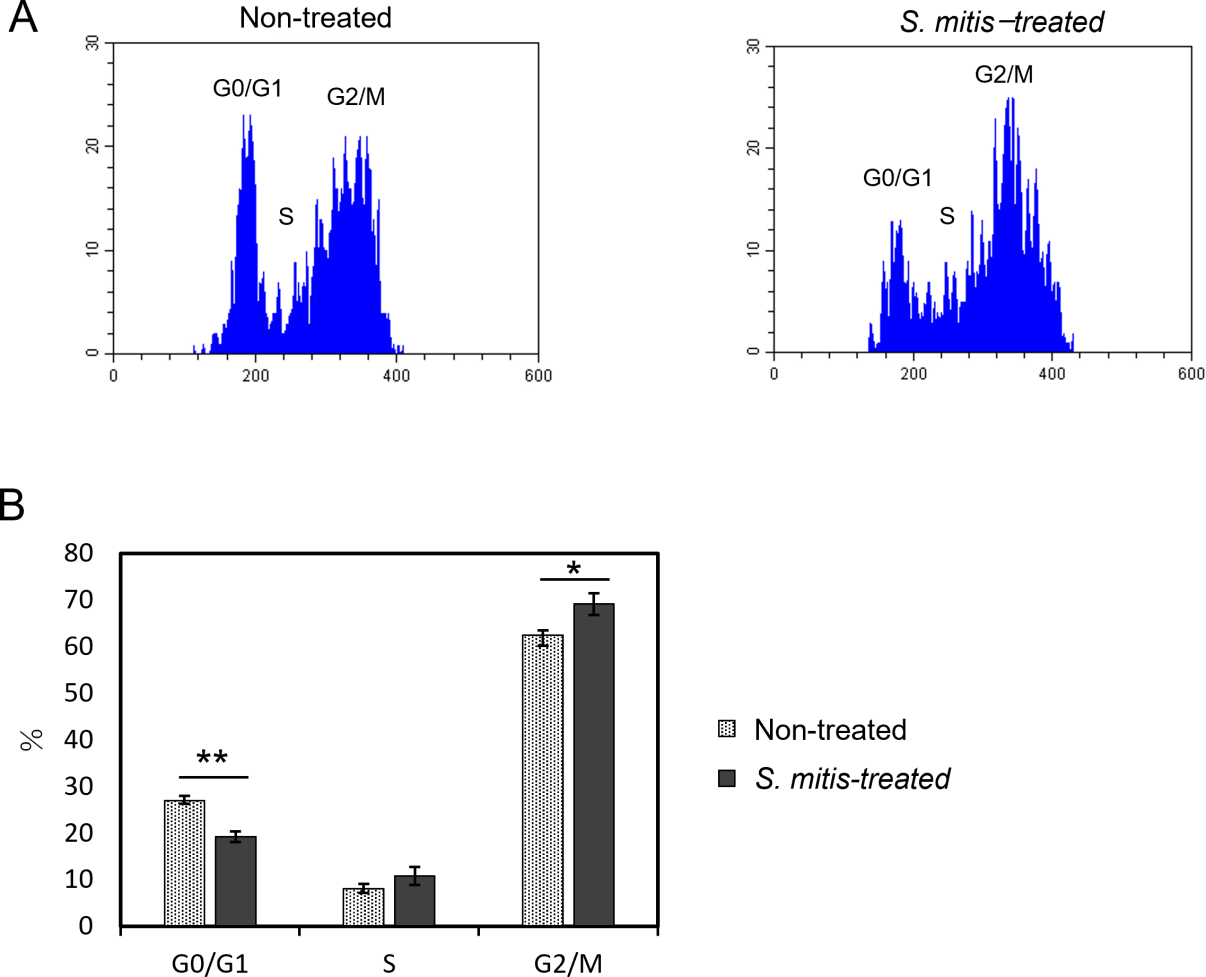
Cell cycle changes of HSC-3 cells treated with *S. mitis*. The cells were stained with propidium iodide (PI) and analyzed using a flow cytometer. **(A)** The DNA content and cell count of the cell population are shown. The vertical axis represents the cell count, and the horizontal axis shows the DNA fluorescence intensity. **(B)** Comparison of the percentage of cells in each region analyzed by flow cytometry. *p < 0.05, **p < 0.01 (Student’s t-test).

In flow cytometry experiments, HSC-3 cells were incubated with a 1.56% dilution of *S. mitis* bacterial suspension (see section 3.2), and changes in the cell cycle were measured. **Figure 4A** shows a histogram of the flow cytometry results of the cell cycle changes induced by *S. mitis*. The percentage of cells in the G0/G1 phase decreased and that in the G2/M phase increased in the *S. mitis-treated* group (**Figure 4B**). As the proliferation of cancer cells was inhibited by *S. mitis*, these bacteria may have inhibited the proliferation of HSC-3 cells by arresting them in the G2/M phase.

### 3.4 Live and dead *S. mitis*, but not secretions of *S. mitis*, inhibit the growth of cancer cells

**Figure 5:**
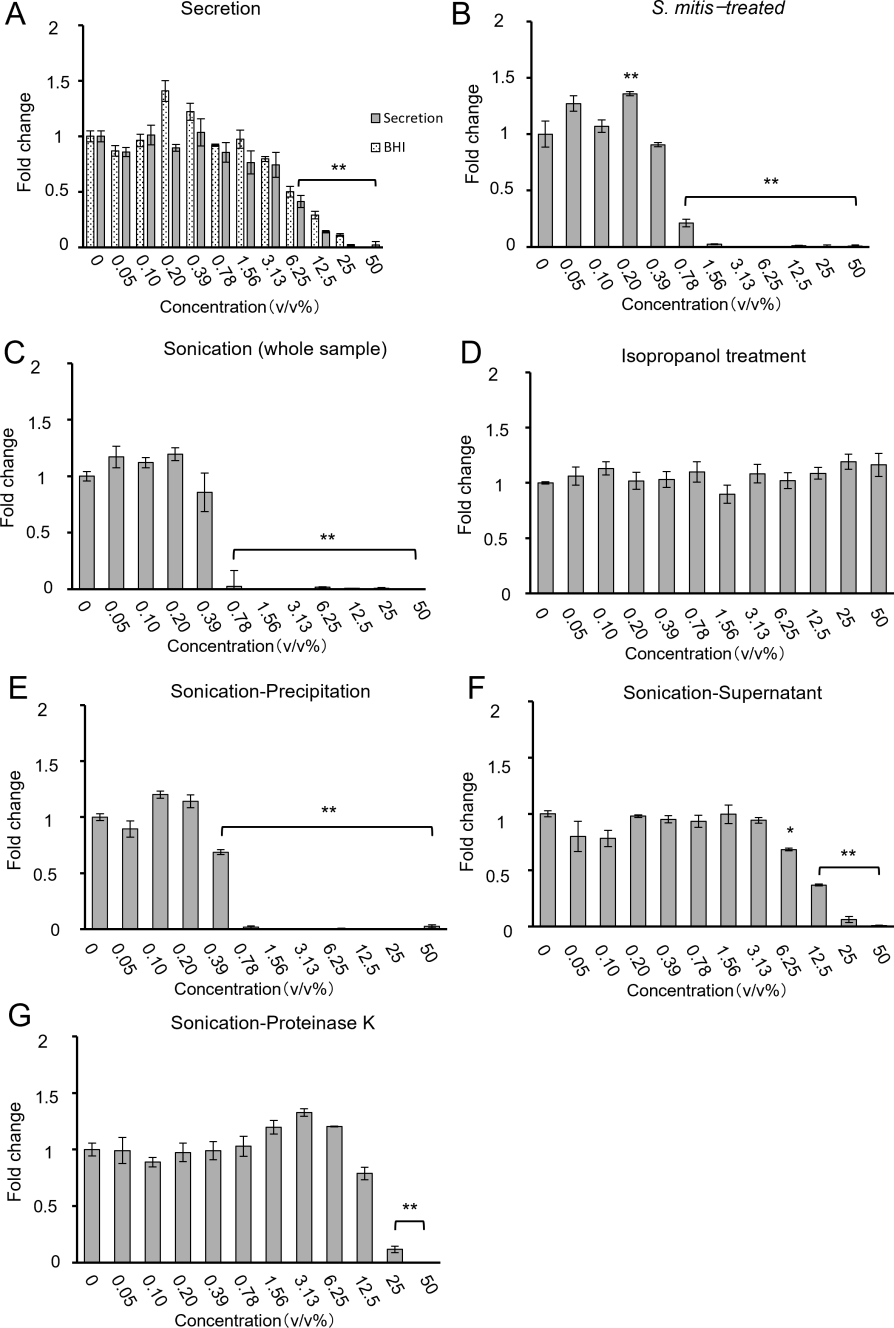
Cell proliferation when processed *S. mitis* (**(A)** secretion, **(B)** intact *S. mitis*, **(C)** sonication (whole sample), **(D)** isopropanol, **(E)** sonication-precipitate, **(F)** sonication-supernatant, and **(G)** sonication-Proteinase K) was added to HSC-3. Cell proliferation was evaluated using the CCK-8 assay (n = 3). The vertical axis shows the concentration of the bacterial solution (v/v%), and the horizontal axis represents the fold change. *p < 0.05, **p < 0.01 (Tukey’s test after one-way analysis of variance).

To identify the mechanism by which *S. mitis* inhibits the growth of HSC-3 cells, samples with various concentrations of live and dead *S. mitis* were added to HSC-3 cells. The supernatant of centrifuged culture media comprised BHI medium and bacterial secretions. The supernatants containing bacterial secretions at concentrations of 12.5% and 25% showed a significant decrease in growth inhibition compared to supernatants containing only BHI. These results suggest that most of the growth-inhibitory effect of the culture supernatant was due to BHI, whereas bacterial secretions had only a minor contribution (**Figure 5A**). The WST-8 results (**Figure 5B**) are similar to those for viable *S. mitis* (section 3.1). Ultrasonically disrupted *S. mitis* samples inhibited cell growth at concentrations of > 0.78%, similar to live *S. mitis* samples (**Figure 5C**). However, chemical disruption of bacteria by isopropanol treatment did not inhibit HSC-3 growth at any concentration (**Figure 5D**).

The ultrasonicated material was separated by centrifugation into cell wall components (precipitate) and bacterial contents (supernatant) to examine their separate effects. After sonication and centrifugation, the precipitate inhibited growth at ≥ 0.78%, as did the viable and sonicated samples (**Figure 5E**). Similarly, the supernatant inhibited cell growth at concentrations of > 12.5%, indicating that supernatants were less effective than the precipitate (**Figure 5F**). This suggests that the cell wall components of *S. mitis* are important for inhibiting cell growth. Cell wall components include lipid bilayers and membrane proteins. To determine which components are particularly important, the proteins were inactivated. Ultrasonically crushed bacteria treated with Proteinase K to degrade proteins achieved cell growth inhibition at concentrations of > 25% (**Figure 5G**). Protein degradation resulted in a decreased growth inhibition, indicating that protein components of the bacterial cell wall affect the growth inhibition of cancer cells. The viability of bacteria after isopropanol treatment and sonication was confirmed using LIVE/DEAD staining (**Supplementary Figure 3**). Based on the above results of the WST-8 assay, the IC80 concentrations for cancer cell inhibition compared to control conditions are summarized in **Table 3**.

**Table 3:** IC80 of HSC-3 cells following the addition of intact *S. mitis* or its components.

Bacterial component	IC80 (v/v%)
Secretion	–
*S. mitis*	0.78
Isopropanol treatment	–
Sonication (whole sample)	0.78
Sonication-precipitation	0.78
Sonication-supernatant	25
Sonication-Proteinase K	25

## 4 Discussion

In the present study, we investigated the effects of major strains of the genus *Streptococcus* on oral cancer cells *in vitro*. We found that *S. mitis* inhibited the growth of oral cancer cells *in vitro*. Furthermore, we observed that *S. mitis* increased the expression of mitosis-related genes in HSC-3 cells and caused cell cycle arrest in the G2/M phase. This activity of *S. mitis* was related to structural bacterial proteins. By matching the absorbance of the culture medium to equalize the number of bacteria, we compared five species, *S. mitis, S. salivarius, S. sanguinis, S. anginosus, and S. mutans*, and found that *S. mitis* had the strongest inhibitory effect on the growth of HSC-3 cells (**Figure 1**). Previous studies have shown that *S. anginosus* inhibits the proliferation of the OSCC cells SCC15 after 16 h of stimulation (Xu et al., 2021) and *S. mitis* reduces the proliferation of OSCC CAL27 cells to less than 50%; *S. sanguinis* inhibits cell proliferation to the same extent as *S. mitis*; and *S. mutans* does not inhibit cell proliferation within 24–72 h stimulation (Baraniya et al., 2020). These results are consistent with many of our findings; however, the lack of similar effects of *S. mitis and S. sanguinis* treatments may be partly due to differences in susceptibility between the two cell lines used in both studies.

Although *S. mitis* can cause bloodstream infections in immunocompromised patients, it is a relatively harmless oral *Streptococcus* (Mitchell, 2011) and a health-related commensal organism. *S. mitis* is most common in the oral cavity of healthy adults with periodontal pockets less than 4 mm deep, and its percentage decreases in patients with periodontitis and periodontal pockets greater than 4 mm (Costalonga and Herzberg, 2014). In relation to oral cancer, *S. mitis* was significantly more frequently detected in the saliva of patients with OSCC (Mager et al., 2005), while a significant decrease in *S. mitis* abundance has been observed as the cancer progresses (Yang et al., 2018). Predictors of these causes include the species specificity of oral colonization by *Streptococcus* (Mager et al., 2005) and changes in the oral microenvironment of patients with advanced tumors (Yang et al., 2018). Our results suggest that *S. mitis* may play a protective role in patients with oral cancer by increasing the frequency of these bacteria in the oral cavity.

To confirm whether *S. mitis* effectively prevents the proliferation of oral cancer cells, we examined HSC-3 and Ca9-22 cells and found that the growth of both cell lines was suppressed by *S. mitis* (**Figure 2**). In a previous study, *S. mitis* was co-cultured with the OSCC cell lines CAL27, SCC25, and SCC4, and growth suppression was confirmed in all cases. Therefore, we can assume that *S. mitis* suppresses the growth of various oral cancer cells (Baraniya et al., 2020). In all experiments in this study, antibiotics were added to the culture medium used for cancer cells; therefore, we believe that abnormal bacterial growth did not occur.

A comprehensive survey of gene expression showed that the number of genes involved in mitotic nuclear division was significantly increased in HSC-3 cells treated with *S. mitis* (**Figure 3A**). In this study, we focused on *DUSP1*, which showed the most significant changes among the specific genes included in the analyzed GO terms. DUSP1 is a dual-specificity phosphatase that regulates mitogen-activated protein kinase (MAPK) activity (Lawan et al., 2013). DUSP1 expression is decreased in OSCC (Tomioka et al., 2006), and DUSP1 downregulation promotes the progression of head and neck squamous cell carcinoma (Zhang et al., 2014). In the present study, *DUSP1* expression was increased in HSC-3 cells whose growth was suppressed by *S. mitis* (**Figure 3B**), suggesting that changes in MAPK signaling by DUSP1 are an important mechanism for growth suppression. According to reports on cell cycle regulation, an increase in DUSP1 expression in gastric cancer leads to a cell cycle arrest in the G1 phase (Li et al., 2003), and it has been reported that DUSP1 expression in cumulus cells is reduced in the G0/G1 phase and increased in the S phase (Fu et al., 2019). However, specific reports regarding the involvement of DUSP1 in the cell cycle of oral cancer cells have not been published. In the future, the effects of increased DUSP1 expression on the growth of oral cancer cells should be investigated.

According to the results of the cell cycle assay (**Figure 4**), the number of HSC-3 cells stimulated with *S. mitis* in the G0/G1 phase decreased, whereas the number of cells in the G2/M phase increased; the proportion of cells in the S phase did not change significantly. This suggests that *S. mitis* induces G2/M phase arrest during the cell cycle progression of HSC-3 cells, thereby inhibiting proliferation. To the best of our knowledge, this is the first report on changes in the cell cycle following *S. mitis* infection of oral cancer cells. Previously, it has been reported that CAL27 cells treated with *F. nucleatum* showed increased proliferation and an increased G2/M phase ratio. Furthermore, when the phosphorylation inhibitor genistein was added, neither the proliferation rate nor the proportion of cells in the G2/M phase increased; however, the detailed mechanism of action is unclear (Li et al., 2024). Thus, *S. mitis* may affect each stage of the cell cycle, but further investigations are required.

To elucidate the mechanism of action underlying the effects of *S. mitis* on HSC-3 cells, we investigated the effects of intact bacteria and bacterial components of *S. mitis* on cancer cells. Previous studies have reported that many of the cytotoxic effects of bacteria are caused by H_2_O_2_ produced by them (Baraniya et al., 2020). Our study findings show that culture supernatants containing bacterial secretions had almost no growth-inhibiting effect (**Figure 5A**). Using a hydrogen peroxide test paper, we confirmed that the hydrogen peroxide concentration in the bacterial solution after resuspending the pellets and sonication was almost the same as that in E-MEM, the culture medium for cancer cells (**Supplementary Figure 4**). The growth-inhibiting effect did not differ when alive *S. mitis* was added, after it had been killed by ultrasound treatment, or when the bacterial cytoplasm content was extracted by centrifugation. Proteinase K degradation of proteins (Ebeling et al., 1974) reduced this inhibitory effect of *S. mitis*, albeit not completely. These findings strongly indicate that structural proteins within *S. mitis* contribute significantly to its anti-proliferative effects on cancer cells. Isopropanol (70%) is used as a disinfectant and is effective against Gram-positive bacteria. Protein denaturation is believed to occur in bacteria treated with isopropanol (Rutala and Weber, 2019). The differences in the effects of protein degradation by Proteinase K and that by isopropanol may provide clues for elucidating the structure of specific types of structural proteins involved in the observed inhibitory effects of *S. mitis*.

Oral cleaning is important in the treatment of periodontal diseases (Loesche and Grossman, 2001). Without periodontal disease, the proportion of *S. mitis* is high in the oral cavity (Costalonga and Herzberg, 2014). Therefore, the fact that *S. mitis* suppresses oral cancer may support the importance of oral cleaning in patients with oral cancer. However, the possibility of unknown adverse effects of *S. mitis* on living organisms cannot be completely ruled out since many effects of *S. mitis*’ functions remain unknown. Because the inhibitory effect of *S. mitis* does not depend on whether the bacteria are alive or dead, it may be preferable to extract and use only the components that are important for the desired result.

This research has some limitations. First, the effects on non-cancer cells have not been investigated. It is necessary to examine whether *S. mitis* exerts cytotoxicity against non-cancerous cells of oral origin and to investigate the effects of *S. mitis* under conditions more similar to those of living organisms by using murine models of oral cancer. Second, the specific components of the bacteria that inhibit cancer cell growth have not been identified. We have shown in this study that protease treatment of the bacterial components attenuates their inhibitory effect, suggesting that structural proteins of the bacteria are important. Therefore, it would be necessary to further elucidate which proteins are important by recombining the genes involved in the expression of structural proteins of *S. mitis*. Third, the mechanism of action against oral cancer has not yet been fully explained. The investigation of receptors and signals on the cancer cell side and the clarification of inhibitory mechanisms may lead to the discovery of new cancer treatments.

## 5 Conclusion

This study revealed that *S. mitis*, a *Streptococcus* species that is the most common oral bacterium, suppresses the growth of oral cancer cells. The mechanism by which *S. mitis* suppresses cancer cell growth mainly involves the growth arrest of mitotic oral cancer cells, as revealed by RNA sequencing and flow cytometry. Our findings suggest that structural proteins of *S. mitis* are involved in these effects. This indicates that *S. mitis* may carry out an important role in the prevention and treatment of oral cancer, and we assume that increasing the number of *S. mitis* in the oral cavity may reduce the risk of oral cancer development.

## Data Availability

The sequencing data have been deposited in the DDBJ Sequence Read Archive under the accession number DRR665257-DRR665262.
