# Supplementary material for: In vitro impact of Streptococcus mitis on the inhibition of oral cancer cell proliferation via mitotic modulation
Source: Front Cell Infect Microbiol. 2025 May 9;15:1524820. doi: 10.3389/fcimb.2025.1524820 (PMC12098639; doi:10.3389/fcimb.2025.1524820)
Supplement: Supplementary file 1 [file Table1.docx]

**Supplementary Material**

***Streptococcus mitis* inhibits the growth of oral cancer cells by affecting mitosis**

**A**

| Gene ID | log2FoldChange | padj | Gene name |
| --- | --- | --- | --- |
| ENSG00000120129 | 1.90 | 0.002 | DUSP1 |
| ENSG00000134222 | 0.65 | 0.018 | PSRC1 |
| ENSG00000164611 | 0.55 | 0.072 | PTTG1 |
| ENSG00000138778 | 0.52 | 0.062 | CENPE |
| ENSG00000121621 | 0.50 | 0.070 | KIF18A |
| ENSG00000255112 | 0.46 | 0.035 | CHMP1B |
| ENSG00000013810 | 0.42 | 0.072 | TACC3 |

B

DUSP1/PSRC1/CHMP1B/CENPE/KIF18A/PTTG1/TACC3/BUB1/NUF2/CCNB1/DLGAP5/CHEK2/NDC80/NUSAP1/ANAPC4/KIF20B/SPAG5/KNSTRN/KIF23/BORA/KIF14/BUB1B/KIF11/TPX2/MAD2L1/CDC20/BIRC5/CDCA8/MZT1/AKAP8L/AURKA/RACGAP1/NCAPG/KIF4A/UBE2S/PLK1/AURKB/KIF2C/CENPF/HIRA/CDC25C/IL1A/NEK2/CCSAP/RCC1/TTK/PCID2/RAD21/SMC4/CEP57L1/CEP192/KIF22

**Supplemental Figure 1.**

**A**

**Supplemental Figure 1.**

**A** Specific gene included in the group of genes related to mitosis and nuclear division.

padj<= 0.1.

**B** Gene involved in increased mitosis and nuclear fission.


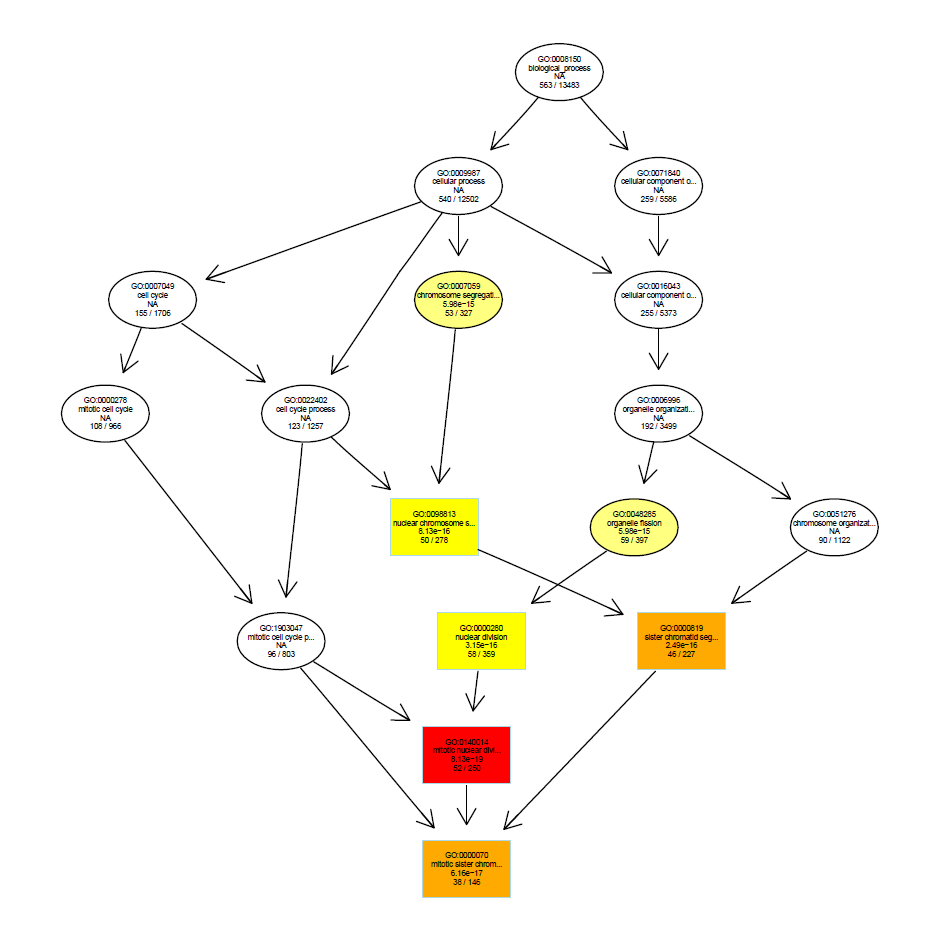


**Supplemental Figure 2.**

Directed acyclic graph (DAG) of the biological processes of GO analysis of genes that were altered in HSC-3 cells. Each node represents a GO term and the boxes show the enrichment levels of the top five most significant GO terms. Darkness of the color indicates the degree of enrichment, with darker colors indicating higher enrichment levels. Each node shows the name of the term and padj value of the enrichment analysis.


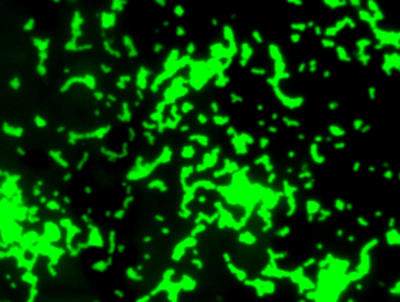

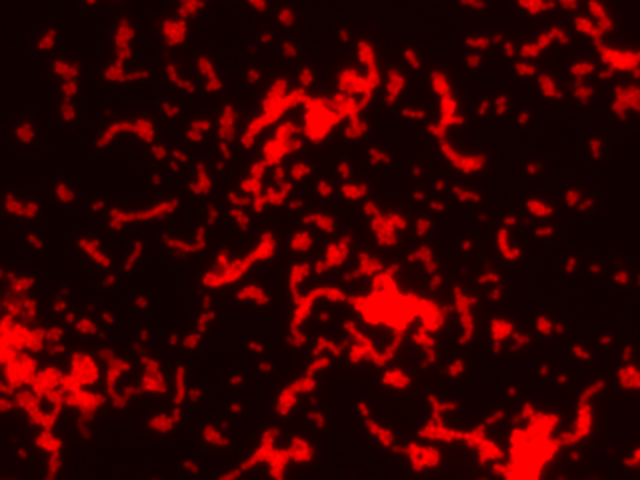

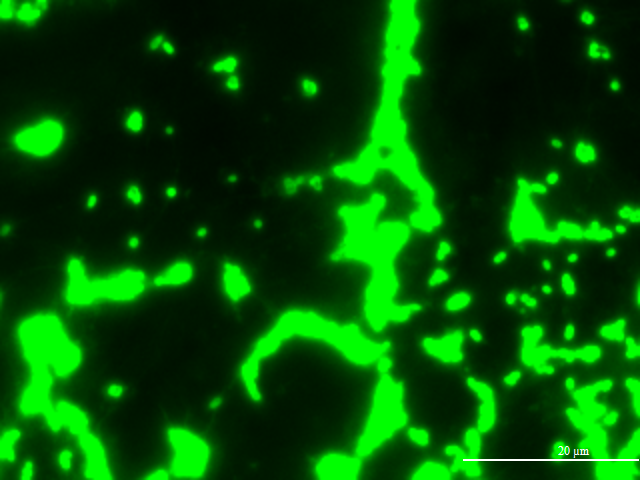

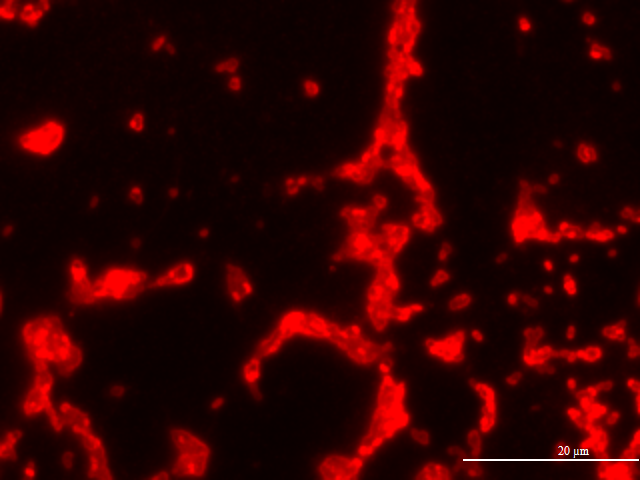

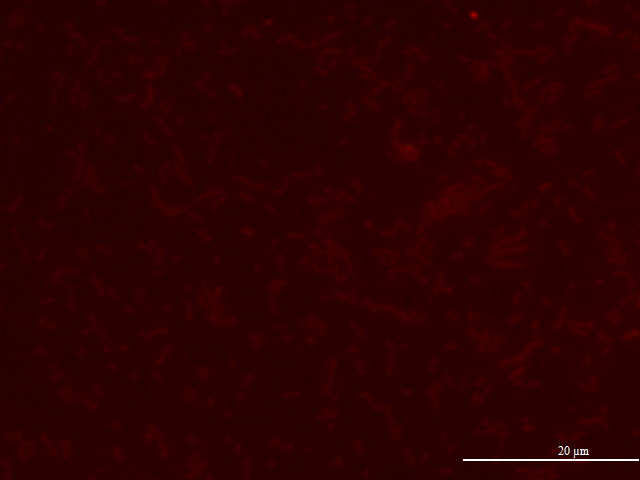

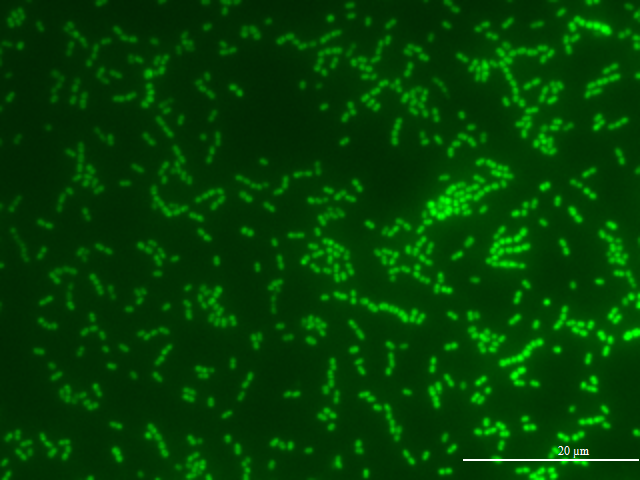


SYTO9

PI

*S. mitis*

Sonication

Isopropanol

**Supplemental Figure 3.**

LIVE/DEAD staining was performed on each processed *S. mitis* before adding it to the cells.

Both live and dead bacteria were stained with SYTO9 and dead bacteria were stained with PI.

**
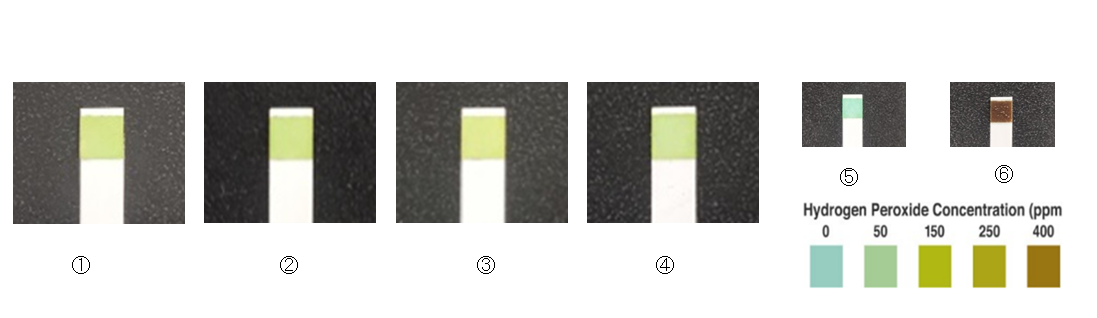

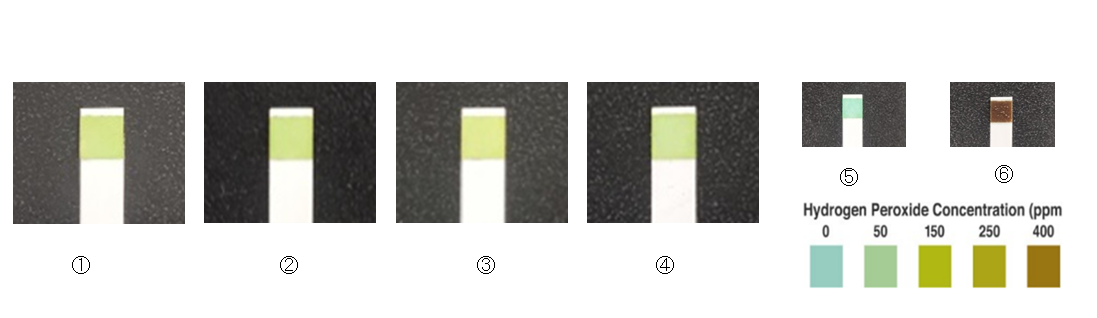

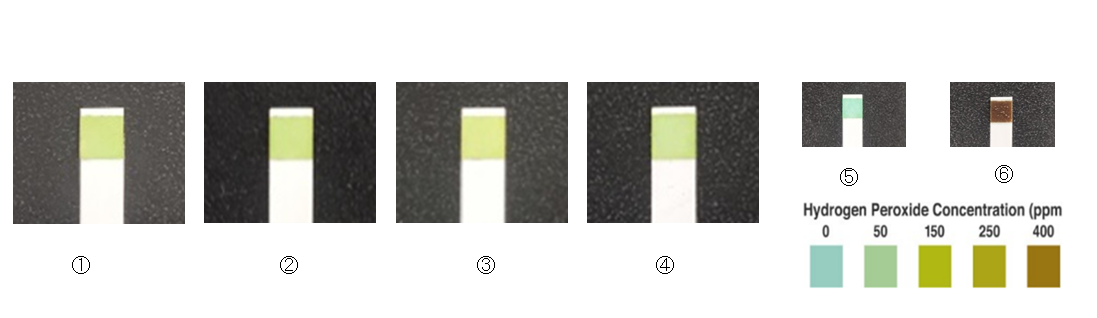
**

⑤

⑥

②

③

④

①

**Supplemental Figure ４.**

Hydrogen Peroxide Test Paper

①*S. mitis* (bacterial solution）② Sonication（whole sample）③Sonication-Precipitation

④E-MEM ⑤RO water（low control）⑥Actril（hydrogen peroxide0.8%　Peracetic acid0.06%）(high control)

Previous research has been conducted on the color changes of test strips at different concentrations (Nakamura et al., 2023).

**References**

Nakamura, Y., Watanabe, K., Yoshioka, Y., Ariyoshi, W., Yamasaki, R. (2023). Persister cell formation and elevated lsra and lsrc gene expression upon hydrogen peroxide exposure in a periodontal pathogen Aggregatibacter actinomycetemcomitans. Microorganisms. 11, 1402. doi: [10.3390/microorganisms11061402](https://doi.org/10.3390/microorganisms11061402).
